# Sustainable production of the drug precursor tyramine by engineered *Corynebacterium glutamicum*

**DOI:** 10.1007/s00253-024-13319-8

**Published:** 2024-10-30

**Authors:** Sara-Sophie Poethe, Nora Junker, Florian Meyer, Volker F. Wendisch

**Affiliations:** Genetics of Prokaryotes, Faculty of Biology and CeBiTec, Bielefeld University, Universitätsstr. 25, 33615 Bielefeld, Germany

**Keywords:** *Corynebacterium glutamicum*, Tyramine, Aromatic amino acid decarboxylase, Sustainable amine production, Plant alkaloids, Pharmaceutical drug precursor

## Abstract

**Abstract:**

Tyramine has attracted considerable interest due to recent findings that it is an excellent starting material for the production of high-performance thermoplastics and hydrogels. Furthermore, tyramine is a precursor of a diversity of pharmaceutically relevant compounds, contributing to its growing importance. Given the limitations of chemical synthesis, including lack of selectivity and laborious processes with harsh conditions, the biosynthesis of tyramine by decarboxylation of l-tyrosine represents a promising sustainable alternative. In this study, the de novo production of tyramine from simple nitrogen and sustainable carbon sources was successfully established by metabolic engineering of the l-tyrosine overproducing *Corynebacterium glutamicum* strain AROM3. A phylogenetic analysis of aromatic-l-amino acid decarboxylases (AADCs) revealed potential candidate enzymes for the decarboxylation of tyramine. The heterologous overexpression of the respective AADC genes resulted in successful tyramine production, with the highest tyramine titer of 1.9 g L^−1^ obtained for AROM3 overexpressing the tyrosine decarboxylase gene of *Levilactobacillus brevis*. Further metabolic engineering of this tyramine-producing strain enabled tyramine production from the alternative carbon sources ribose and xylose. Additionally, up-scaling of tyramine production from xylose to a 1.5 L bioreactor batch fermentation was demonstrated to be stable, highlighting the potential for sustainable tyramine production.

**Key points:**

• *Phylogenetic analysis revealed candidate l-tyrosine decarboxylases*

• C. glutamicum* was engineered for *de novo* production of tyramine*

• *Tyramine production from alternative carbon substrates was enabled*

**Supplementary information:**

The online version contains supplementary material available at 10.1007/s00253-024-13319-8.

## Introduction

Tyramine is a primary amine that can be found in bacteria, plants, and animals. Recently, there has been a notable increase in interest across various industries with regard to its diverse applications. In addition to its use as a monomer for the production of high-performance thermoplastics (Chen et al. 2023), tyramine has been shown to improve the wettability and formation of stable cell–matrix interactions of alginate-based hydrogels, rendering them promising biomaterials for tissue engineering (Schulz et al. 2019). Moreover, tyramine serves as a precursor to a variety of compounds that hold potential for use in pharmaceutical and industrial applications. For instance, tyrosol that can be derived from tyramine has anti-inflammatory effects (Giovannini et al. 2002) and is itself a precursor for several health-promoting compounds, including β-blockers and the neuroprotective salidroside (Ippolito and Vigmond 1988; Yu et al. 2011; Torrens-Spence et al. 2018). Furthermore, *N*-acetyltyramine has been demonstrated to exhibit activity against multidrug-resistant pathogens and is a putative new antiplatelet drug due to its inhibitory effect on factor Xa (Lee et al. 2017; Driche et al. 2022). Dopamine, another attractive derivative of tyramine, is a neurotransmitter. Furthermore, it forms polydopamines, which have applications in biotechnology for reversible binding of enzymes and other biomolecules. Additionally, polydopamines have been demonstrated to delay fouling when applied as coatings to membranes used for water purification (Lee et al. 2009; McCloskey et al. 2010).

The chemical synthesis of primary amines can be achieved through the catalytic hydrogenation of nitriles. However, a lack of selectivity towards the desired amine often results in the formation of secondary or tertiary amines, as well as other undesired side products. Moreover, chemical tyramine synthesis is laborious, comprising multiple synthesis steps, and requires harsh conditions rendering it environmentally unfavorable (Hegedűs and Máthé 2005; McAllister et al. 2018). Conversely, the biotechnological production of tyramine from l-tyrosine can be achieved under mild conditions by selective pyridoxal-5′phosphate dependent decarboxylation of aromatic l-amino acids by AADCs (EC 4.1.1.28; Li et al. 2012). Therefore, whole-cell biotransformation utilizing AADCs for tyramine production represents a desirable alternative to chemical synthesis, as the enzymatic decarboxylation of l-tyrosine occurs at ambient pH and temperature. Furthermore, biotransformation allows for a high degree of selectivity, thereby offering more cost-efficient production processes compared to the chemical synthesis (Lin and Tao 2017; Sheldon and Woodley 2018). However, whole-cell biotransformation necessitates an external supply of l-tyrosine and its efficient import into the cells (Zhu et al. 2020). To circumvent this limitation, de novo biosynthesis of tyramine from simple nitrogen and carbon sources can be employed. In bacteria, l-tyrosine is synthesized via the shikimate pathway (Chávez-Béjar et al. 2012). Metabolic engineering can extend the biosynthesis of l-tyrosine by its subsequent decarboxylation, thus, enabling the de novo production of tyramine from simple starting materials such as ammonia and glucose or more sustainable carbon sources such as xylose or arabinose, which are prevalent in lignocellulosic agricultural waste products (Aristidou and Penttilä 2000).

*Corynebacterium glutamicum* is an optimal host for metabolic engineering aimed at tyramine production since it is a well-established workhorse in the industry, utilized for the production of millions of tons of amino acids per year (Wendisch 2020). It exhibits the capacity to utilize a diverse range of carbon sources, encompassing various sugars, such as glucose, fructose, maltose, sucrose, and ribose (Blombach and Seibold 2010), as well as organic acids, including acetate and lactate (Gerstmeir et al. 2003; Stansen et al. 2005). Furthermore, it has been engineered to utilize various alternative carbon sources, including arabinose (Kawaguchi et al. 2008; Schneider et al. 2011), xylose (Gopinath et al. 2011; Meiswinkel et al. 2013), and glycerol (Rittmann et al. 2008). *C. glutamicum* has been genetically engineered for the production of high-value active ingredients for food, feed, human well-being and health (Wolf et al. 2021). For example, various aromatic compounds, including l-phenylalanine (Zhang et al. 2015), l-tryptophan (Bampidis et al. 2019), *N*-methylphenylalanine (Kerbs et al. 2021), halogenated tryptophan derivatives (Veldmann et al. 2019), indole (Mindt et al. 2022), and protocatechuic acid (Okai et al. 2016) can be obtained in fermentative processes using engineered *C. glutamicum* strains.

**Fig. 1:**
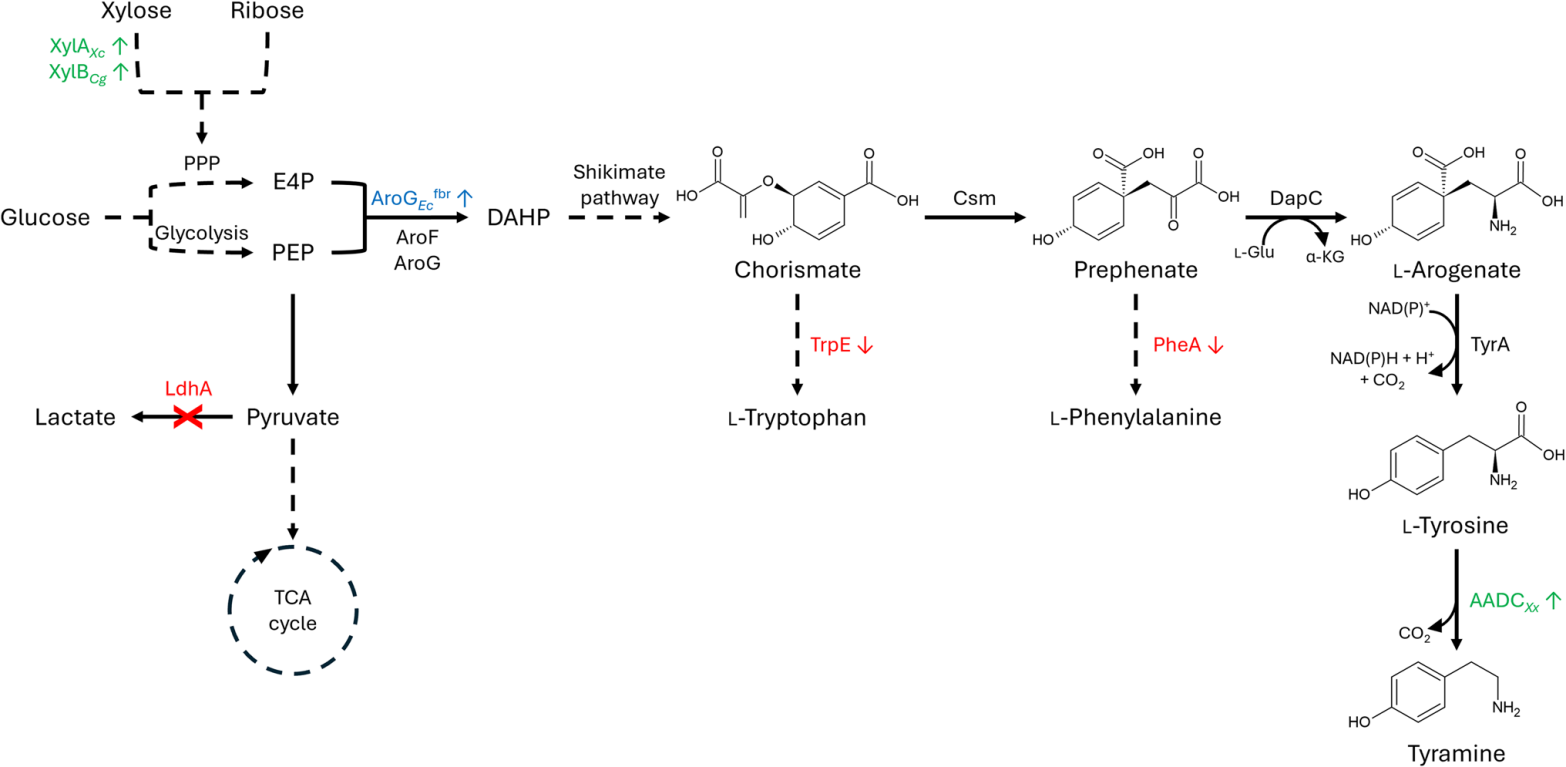
Scheme of metabolic engineering of *C. glutamicum* AROM3 for tyramine production. Continuous arrows indicate single reaction steps, while dashed arrows indicate multiple reaction steps. Genetic modifications of AROM3 are depicted by colored enzyme names, whereby a blue arrow up indicates overexpression, red arrows down indicate reduced expression by start codon exchange from ATG to TTG, and an arrow marked with a red cross indicates gene deletion. Overexpression of genes for tyramine production from alternative carbon sources are represented by green enzyme names and arrows pointing up. AADC_*Xx*_: aromatic-l-amino acid decarboxylase from different organisms, AroF and AroG: DAHP synthase, AroG_*Ec*_^fbr^: feedback-resistant DAHP synthase from *E. coli*, Csm: chorismate mutase, DapC: *N*-succinyl-aminoketopimelate aminotransferase, DAHP: 3-deoxy-d-arabinoheptulosonate-7-phosphate, E4P: erythrose 4-phosphate; LdhA: lactate dehydrogenase, NAD(P): nicotinamide adenine dinucleotide (phosphate), PEP: phosphoenolpyruvate, PheA: prephenate dehydratase, PPP: pentose phosphate pathway, TrpE: anthranilate synthase, TyrA: arogenate dehydrogenase, XylA: xylose isomerase, XylB: xylulose kinase

In this study, the l-tyrosine synthesis pathway in *C. glutamicum* was extended by l-tyrosine decarboxylation to enable de novo biosynthesis of tyramine. Previously, metabolic engineering has resulted in the l-tyrosine overproducing strain AROM3, achieving an l-tyrosine titer of 3.1 g L^−1^ (Kurpejović et al. 2023). Moreover, the heterologous overexpression of AADC genes from *Bacillus* *atrophaeus*, *Clostridium* *sporogenes*, and *Ruminococcus gnavus* in an l-tryptophan overproducing *C. glutamicum* strain enabled the de novo production of tryptamine (Kerbs et al. 2022). To identify potential AADCs active with l-tyrosine, a phylogenetic analysis was conducted and four candidate AADC genes were selected and overexpressed in the l-tyrosine overproducing strain AROM3 to facilitate tyramine production (Fig. 1). Finally, the substrate spectrum of the optimal tyramine-producing strain was expanded to encompass alternative carbon sources, thereby facilitating sustainable tyramine production.

## Material and methods

### Bacterial strains and growth conditions

Bacterial strains and plasmids used in this study are listed in Table 1. *Escherichia coli* and *C. glutamicum* were cultivated at 37 °C and 180 rpm or at 30 °C and 120 rpm, respectively, in lysogeny broth (LB, Bertani 1951) medium or brain–heart infusion (BHI) medium supplemented with 91 g L^−1^ sorbitol. When appropriate, the medium was supplemented with kanamycin 25 µg mL^−1^ and tetracycline 5 µg mL^−1^.

**Table 1:** Strains and plasmids used in this work

Strain	Relevant characteristics	Antibiotic resistance	Supplements	Source
*E. coli* DH5α	*supE44*Δ*lacU169* (Φ*80dlacZ*Δ*M15*) *hsdR17 recA1 endA1 gyr96 thi-1 relA1*	-	-	Hanahan (1983)
AROM3	C1* Δ*ldhA* Δ*vdh*::P_*ilvC*_-*aroG*_*Ec*_^D146N^ *trpE*^M1L^ *pheA*^M1L^	-	l-Phenylalanine	Kurpejović et al. (2023)
AROM3 EV	AROM3 harboring pECXT99A	Tet^R^	l-Phenylalanine, IPTG	This study
AROM3 *aaDC*_*Ba*_	AROM3 harboring pECXT-P*syn*-*aaDC*_*Ba*_	Tet^R^	l-Phenylalanine	This study
AROM3 *aaDC*_*Cs*_	AROM3 harboring pECXT-P*syn-aaDC*_*Cs*_	Tet^R^	l-Phenylalanine	This study
AROM3 *aaDC*_*Rg*_	AROM3 harboring pECXT-P*syn*-*aaDC*_*Rg*_	Tet^R^	l-Phenylalanine	This study
AROM3 *tdc*_*Lb*_	AROM3 harboring pECXT99A-*tdc*_*Lb*_	Tet^R^	l-Phenylalanine, IPTG	This study
AROM3 *tdc*_*Lb*_* xylA*_*Xc*_*B*_*Cg*_	AROM3 harboring pECXT99A-*tdc*_*Lb*_ and pVWEx1-*xylA*_*Xc*_*-xylB*_*Cg*_	Tet^R^, Kan^R^	l-Phenylalanine, IPTG	This study
Plasmid				
pECXT99A	*C. glutamicum*/*E. coli* shuttle vector for inducible overexpression, P*trc*, *lacI*^q^, pGA1 mini oriV_*Cg*_, pMB1 oriV_*Ec*_	Tet^R^	IPTG	Kirchner and Tauch (2003)
pECXT-P*syn*-*aaDC*_*Ba*_	pECXT-P*syn* derivative for overexpression of codon-harmonized *aaDC* from *B. atrophaeus* with an optimized RBS	Tet^R^	-	Kerbs et al. (2022)
pECXT-P*syn-aaDC*_*Cs*_	pECXT-P*syn* derivative for overexpression of codon-harmonized *aaDC* from *C. sporogenes* with an optimized RBS	Tet^R^	-	Kerbs et al. (2022)
pECXT-P*syn-aaDC*_*Rg*_	pECXT-P*syn* derivative for overexpression of codon-harmonized *aaDC* from *R. gnavus* with an optimized RBS	Tet^R^	-	Kerbs et al. (2022)
pECXT99A-*tdc*_*Lb*_	pECXT99A derivative for overexpression of codon-harmonized *tdc* from *L. brevis* with an optimized RBS	Tet^R^	IPTG	This study
pVWEx1-*xylA*_*Xc*_*B*_*Cg*_	Derivative from pVWEx1 for inducible overexpression of *xylA* from *Xanthomonas campestris* and *xylB* from *C. glutamicum*	Kan^R^	IPTG	Meiswinkel et al. (2013)

Growth and production experiments with *C. glutamicum* strains were performed under selective conditions in 100 mL baffled shake flasks with 10 mL CGXII minimal medium (Eggeling and Bott 2005) containing 40 g L^−1^ glucose as carbon source, if not mentioned otherwise, and supplemented with 0.5 mM l-phenylalanine (Kurpejović et al. 2023). The CGXII minimal medium was inoculated to an initial optical density at a wavelength of 600 nm (OD_600_) of 1 and growth was monitored for 72 h by measurement of the OD_600_ using a V-1200 spectrophotometer (VWR, Radnor, PA, USA). OD_600_ was converted to g L^−1^ cell dry weight (CDW) by multiplication with the experimentally determined conversion factor 0.353. Gene expression of the plasmids pECXT99A and pVWEx1 and their derivatives was induced by addition of 1 mM isopropyl-β-d-1-thiogalactopyranoside (IPTG). To assess possible toxic effects of tyramine, *C. glutamicum* AROM3 was cultivated in CGXII minimal medium containing tyramine at different concentrations.

### Molecular genetic techniques and strain construction

All primers used for DNA amplification and sequencing (Supplementary Table S1) were purchased from Sigma-Aldrich (Ulm, Germany). Plasmid isolation and DNA purification were performed using the GeneJET Plasmid Miniprep Kit (Thermo Fisher Scientific, Schwerte, Germany) and the NucleoSpin Gel and PCR Clean-up Kit (Macherey–Nagel GmbH & Co. KG, Düren, Germany) according to the manufacturer’s instructions. DNA concentrations were measured using the NanoDrop 1000 Spectrophotometer (Thermo Fisher Scientific Inc., Waltham, MA, USA) at 260 nm.

The *tdc* gene from *L. brevis* (Genbank: JX204286) was codon-harmonized for *C.* *glutamicum* (Haupka 2020, Supplementary Table S2) and constructed by gene synthesis by Twist Bioscience (South San Francisco, CA, USA). Using an Allin™ HiFi DNA Polymerase (highQu GmbH, Kraichtal, Germany), the *tdc*_*Lb*_ was amplified with the primers tdc_pECXT99A_fw and tdc_pECXT99A_rv adding pECXT99A overhangs as well as an optimized ribosome binding site (RBS) calculated using the Salislab software (Reis and Salis 2020, Supplementary Table S2). The amplified gene was cloned into the EcoRI linearized pECXT99A-plasmid (New England Biolabs, Frankfurt, Germany) via Gibson assembly (Gibson et al. 2009). Chemocompetent *E. coli* DH5α were prepared by the CaCl_2_ method and transformed with the pECXT99A-*tdc*_*Lb*_ by performing a heat shock at 42 °C (Sambrook and Russel 2001). The correct sequence of the constructed plasmid was verified by sequencing using the appropriate oligonucleotides (Supplementary Table S1).

For the construction of tyramine producing *C. glutamicum* strains, electrocompetent *C. glutamicum* AROM3 cells were prepared and transformed by electroporation followed by a heat shock at 46 °C with the plasmids for the overexpression of the AADC genes (Eggeling and Bott 2005).

### Quantification of aromatic compounds and carbohydrates by HPLC analysis

Extracellular glucose, xylose, and ribose as well as l-phenylalanine, l-tyrosine, and l-tryptophan, and their corresponding amines were quantified using the Agilent 1200 HPLC system (Agilent Technologies Deutschland GmbH, Waldbronn, Germany). Cell culture samples were vortexed vigorously, diluted to the linear range of the corresponding detector, and centrifuged at 20,238 × g and room temperature for 10 min. The supernatant was subjected to HPLC analysis and the resulting data was evaluated with OpenLab CDS (Agilent Technologies Inc., Santa Clara, CA, USA).

For the quantification of the aromatic amino acids and amines, 100 μM cadaverine was added as an internal standard to each sample, and an automatic pre-column derivatization with *o*-phthalaldehyde was performed (Jones and Gilligan 1983). 5 µL sample was injected into a reversed-phase system consisting of a 40 × 4 mm LiChrospher 100 RP18 EC-5 μm pre-column (CS-Chromatographie Service GmbH, Langerwehe, Germany) and a 125 × 4 mm LiChrospher 100 RP18 EC-5 μm (CS-Chromatographie Service GmbH, Langerwehe, Germany) main column. As mobile phase, 0.25% (v/v) sodium acetate, pH 6.0 (solvent A), and methanol (solvent B) were used applying the gradient and flow rates stated in Supplementary Table S3. Detection was carried out with a fluorescence detector (FLD G1321A, 1200 series, Agilent Technologies) with excitation and emission wavelength of 230 nm and 450 nm, respectively.

Carbohydrates were analyzed on a system consisting of a 40 × 8 mm organic acid resin pre-column and a 300 × 8 mm organic acid resin main column with 10 μm particle size (CS-Chromatographie Service GmbH, Langerwehe, Germany). 5 μL sample was injected into the system, and separation was performed at a column temperature of 60 °C using 5 mM sulfuric acid as mobile phase at a constant flow rate of 0.8 mL min^−1^ for 17 min. Detection was performed using a refractive index detector (RID Detector G1362A, Agilent Technologies Deutschland GmbH, Waldbronn, Germany). Pure substances were purchased as standards for identification and quantification.

### Bioreactor batch-mode cultivation

Batch fermentations were conducted in glass bioreactors with a total volume of 3.7 L and a stirrer/reactor diameter ratio of 0.39 (KLF, Bioengineering AG, Wald, Switzerland). Two six-bladed Rushton turbines were positioned on the stirrer axis at heights of 6 cm and 12 cm, accompanied by a mechanical foam breaker at 22 cm.

First precultures of AROM3 *tdc*_*Lb*_ and AROM3 *tdc*_*Lb*_* xylA*_*Xc*_*B*_*Cg*_ were cultivated as described above under selective conditions in 50 mL LB medium containing 10 g L^−1^ glucose or xylose, respectively, for 8 h. Second precultures were performed in 200 mL CGXII minimal medium supplemented with 40 g L^−1^ glucose or xylose, 1 mM l-phenylalanine, 1 mM l-tryptophan, and respective antibiotics. They were inoculated with the first precultures to an OD_600_ of 1 or 3, respectively, and cultivated overnight. For batch fermentations, 1.5 L of CGXII minimal medium without MOPS, containing the same supplements as the second precultures, was inoculated to an initial OD_600_ of 1.

Throughout fermentation, the temperature was kept constant at 30 °C and a steady air flow of 0.3 NL min^−1^ was sustained with a ring sparger. The stirrer speed was automatically adjusted between 400 and 1500 rpm to maintain the relative dissolved oxygen saturation (rDOS) at 30%. The pH was kept constant at 7.0 ± 0.1 by the automatic addition of 10% (v/v) H_3_PO_4_ or 25% (v/v) NH_3_. Foaming was reduced by the addition of antifoam 204 controlled by an antifoam probe. Samples were taken using an autosampler and stored at 4 °C until analysis.

### Phylogenetic analysis of aromatic amino acid decarboxylases

The amino acid sequence of the Tdc_*Lb*_ was subjected to a PSI-BLAST search (Altschul et al. 1997) against the UniProtKB/Swiss-Prot database (The UniProt Consortium 2021) using the BLOSUM62 matrix and an e-value cut-off of 0.001. The resulting list of proteins with high sequence similarity was then aligned with Clustal Omega using the default iteration settings (Sievers et al. 2011). With these alignments, a phylogenetic tree was constructed using IQ-tree v1.6.12 (Trifinopoulos et al. 2016). The parameters used were “-bb 1000 -alrt 1000”. The enzyme groups were labeled and the phylogenetic tree was visualized using Interactive Tree of Life (iTOL) v6.9 (Letunic and Bork 2021).

## Results

### Assessing the suitability of *C. glutamicum* for tyramine production

Several bacteria, including coryneform bacteria, have been reported to possess the ability to degrade tyramine through the utilization of amine oxidases (Leuschner et al. 1998). To determine whether tyramine is degraded by *C. glutamicum* and, at the same time, to evaluate the influence of tyramine on its growth, *C. glutamicum* AROM3 was grown in CGXII minimal medium with 40 g L^−1^ glucose as sole carbon source in the presence of 0–50 mM tyramine for 48 h. Samples were taken from the cultures containing 10 mM tyramine at the beginning and the end of cultivation, and tyramine concentrations were determined via HPLC measurement.

**Fig. 2:**
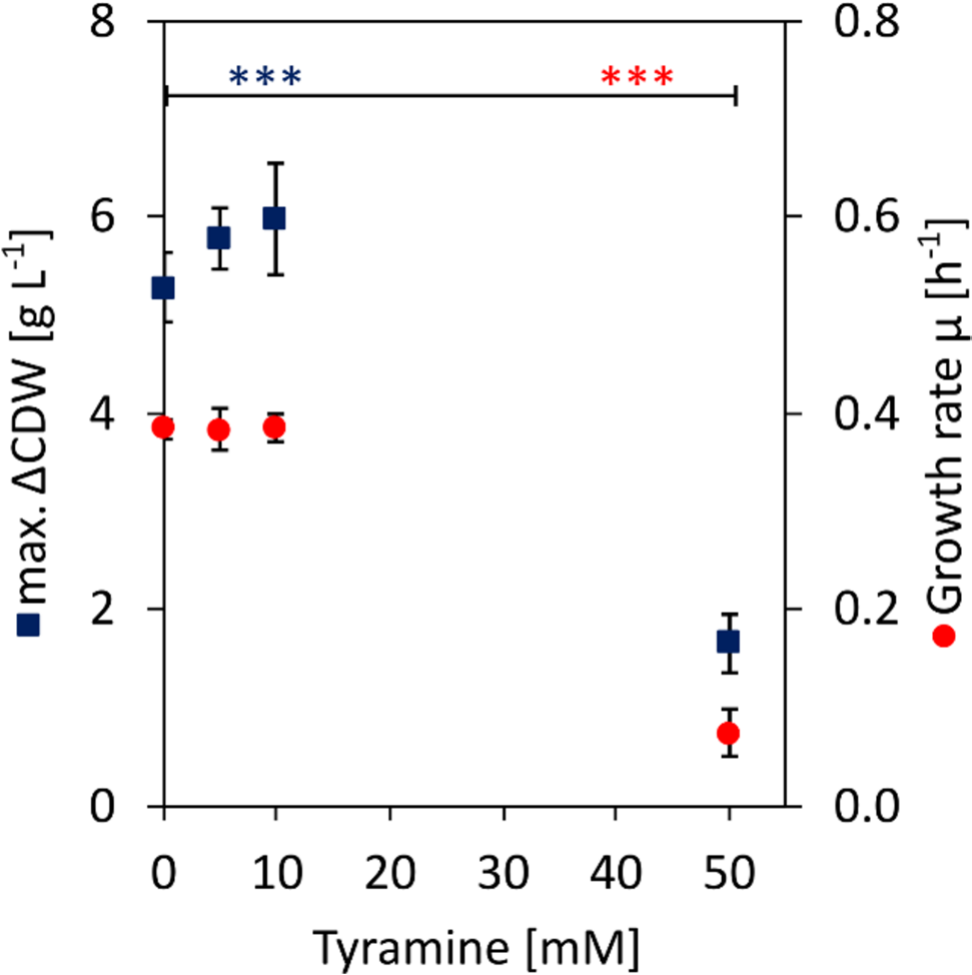
Influence of tyramine on the growth of *C. glutamicum* AROM3. Maximum ΔCDW (dark blue squares) and specific growth rate (red circles) of AROM3 cultivated in 10 mL CGXII minimal medium supplemented with 40 g L^−1^ glucose, 0.5 mM l-phenylalanine, and 0–50 mM tyramine for 48 h. Values and error bars represent means and standard deviations of triplicate cultivations. Significance was calculated with a two-sided Student’s *t*-test with ***: *p* < 0.001. No significant differences in the growth behavior were obtained between cultivations with 0, 5, and 10 mM tyramine

The concentration of tyramine did not decrease within 48 h of cultivation (Supplementary Fig. S1), indicating that AROM3 does not degrade tyramine. Exposure to tyramine concentrations up to 10 mM had no significant effect on the growth of AROM3 in glucose containing minimal medium (Fig. 2). However, in the presence of 50 mM tyramine, the growth rate was significantly reduced and a maximum ΔCDW of only about 30% was reached compared to the culture without tyramine supplementation. These results indicated that high concentrations of tyramine inhibit the growth of AROM3. In summary, the robustness of AROM3 to tyramine concentrations of up to 10 mM and the fact that this strain does not degrade tyramine renders it a suitable candidate for metabolic engineering aimed at the large-scale production of tyramine.

### Phylogenetic analysis of decarboxylases

**Fig. 3:**
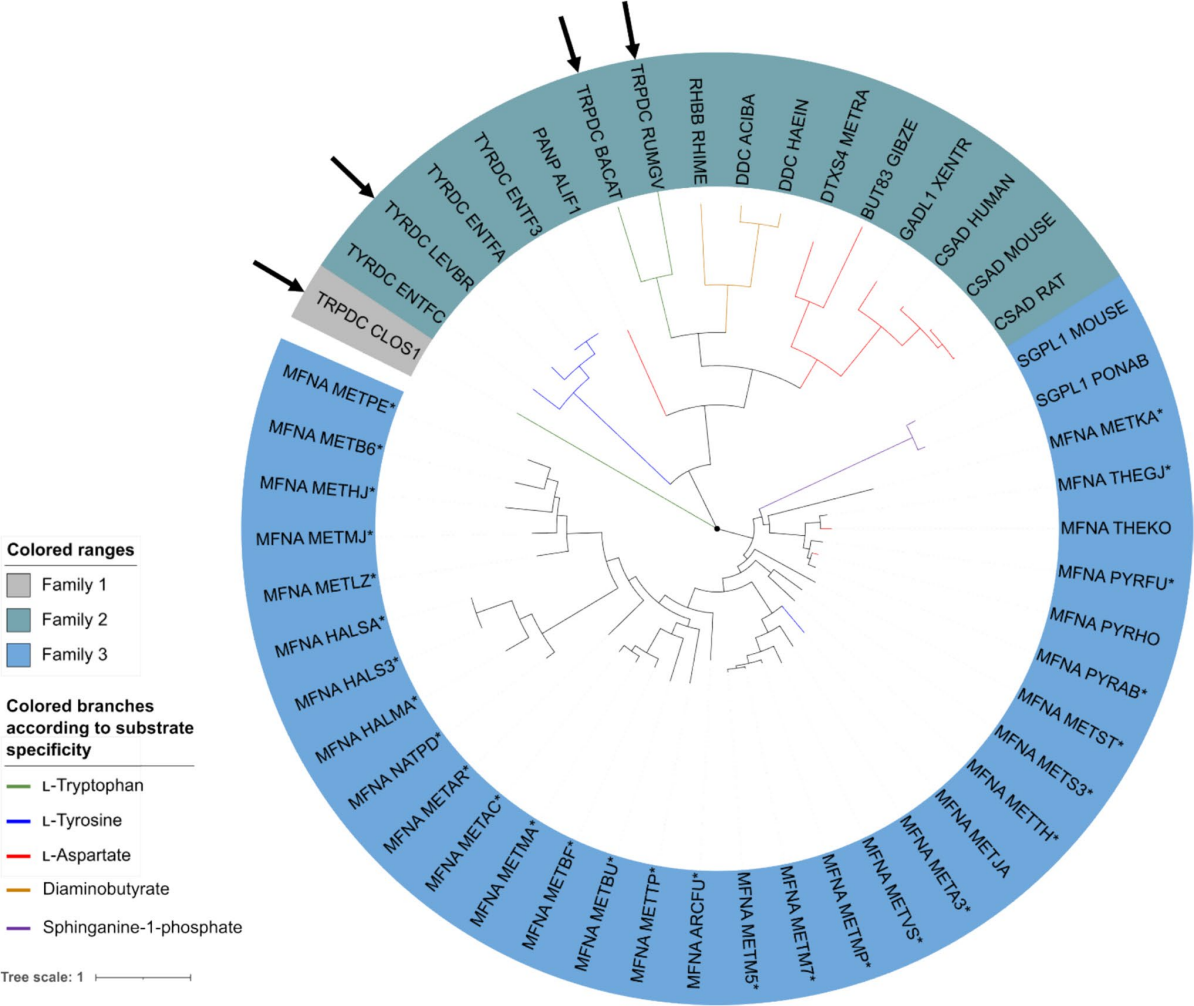
Phylogenetic tree of amino acid decarboxylases divided into three major families. Enzymes are labeled with their annotation and organism as listed in Supplementary Table S4. Branches of characterized decarboxylases were colored according to their substrate specificity. Putative l-tyrosine/l-aspartate decarboxylases are marked with an asterisk. AADCs chosen as candidates for the production of tyramine in AROM3 are highlighted by arrows

Tyramine arises from l-tyrosine by decarboxylation. However, *C. glutamicum* is not known to secrete tyramine and inspection of its genome did not indicate candidate genes encoding l-tyrosine decarboxylases. Similarly, l-tryptophan decarboxylases are absent from the *C. glutamicum* genome and de novo tryptamine production was only achieved via introduction of heterologous aromatic-l-amino acid decarboxylase (AADCs, EC 4.1.1.28) genes from one of the three microorganisms *Bacillus atrophaeus*, *Clostridium sporogenes*, or *Ruminococcus gnavus* in an l-tryptophan overproducing *C. glutamicum* strain (Kerbs et al. 2022). Since these three AADCs accept all three aromatic proteinogenic amino acids as substrates (Williams et al. 2014; Choi et al. 2021), their utilization for tyramine production was an obvious choice. However, these enzymes prefer l-tryptophan over l-tyrosine as substrate. Thus, a phylogenetic analysis was conducted to find related enzyme candidates that might be more specific for l-tyrosine. The phylogenetic analysis depicted in Fig. 3 revealed that the homologous proteins identified by PSI-BLAST can be classified into three major families. Family 1 is unique in that it contains only the AADC from *C. sporogenes* (Williams et al. 2014). By contrast, family 2 contains a diversity of well-annotated decarboxylases, including the two AADCs from *B. atrophaeus* and *R. gnavus*. Therefore, we focused on this family of enzymes to find alternative AADCs for tyramine production. Each branch of family 2 consists of decarboxylases with the same substrate specificity. One of these branches, colored in blue, comprises l-tyrosine decarboxylases (Tdcs, EC 4.1.1.25) from *Enterococcus faecalis* and *Levilactobacillus brevis*. The Tdcs from *E. faecalis* exhibit high activity for l-tyrosine, but can also accept l-phenylalanine as a substrate (Liu et al. 2014). In contrast, the Tdc from *L. brevis* (Tdc_*Lb*_) is specific for l-tyrosine and does not decarboxylate l-tryptophan or l-phenylalanine (Moreno-Arribas and Lonvaud-Funel 2001). In addition, Tdc_*Lb*_ is reported to have a lower K_*m*_ value for l-tyrosine and an approximately 30-fold higher V_*max*_ value compared to *E. faecalis* Tdcs (Zhang and Ni 2014; Liu et al. 2014).

Family 3 includes an l-tyrosine decarboxylase from *Methanocaldococcus jannaschii* (MFNA METJA). However, since this bacterium is thermophilic and its decarboxylase exhibits activity at 70 °C (Kezmarsky et al. 2005), it is not a suitable candidate for in vivo tyramine production in *C. glutamicum*, which is cultivated at its temperature optimum of 30 °C. Besides this enzyme, family 3 consists mostly of enzymes that have not yet been characterized and are therefore annotated as putative l-tyrosine/l-aspartate decarboxylases based on sequence similarity, many of which also originate from thermophiles.

Taken together, besides the AADCs from *C. Sporogenes*, *B.* *atrophaeus*, and *R. gnavus*, we chose the Tdc from *L.* *brevis* as a promising enzyme for tyramine production in AROM3.

### Testing of AADCs for fermentative tyramine production

To conduct a comparative analysis of tyramine production, the candidate decarboxylase gene *tdc*_*Lb*_ and the three AADC genes *aaDC*_*Ba*_, *aaDC*_*Cs*_, and *aaDC*_*Rg*_ previously used for tryptamine production (Kerbs et al. 2022) were overexpressed in the l-tyrosine producing strain AROM3. For this, pECXT-plasmid derivatives carrying either one of the four decarboxylase genes codon-harmonized for *C. glutamicum* or no insert as a negative control were used to transform the strain AROM3. The resulting strains were named AROM3 *tdc*_*Lb*_, AROM3 *aaDC*_*Ba*_, AROM3 *aaDC*_*Cs*_, AROM3 *aaDC*_*Rg*_, and AROM3 EV and were cultivated in glucose containing minimal medium for 72 h.

**Fig. 4:**
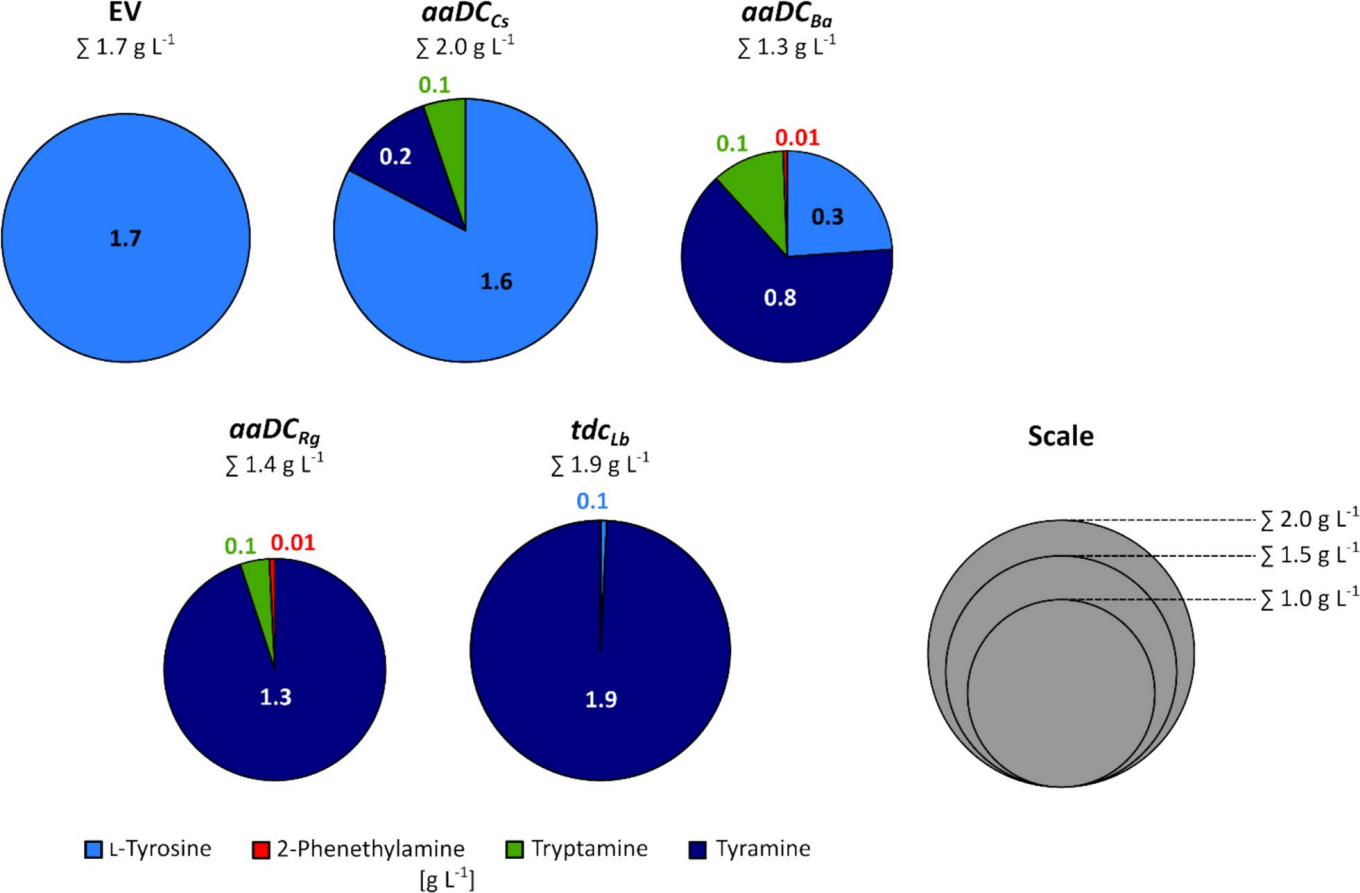
Production of l-tyrosine (light blue), 2-phenethylamine (red), tryptamine (green), and tyramine (dark blue) by *C. glutamicum* AROM3 strains overexpressing different decarboxylase genes. AROM3 strains carrying the plasmids pECXT99A, pECXT99A-*tdc*_*Lb*_, or pECXT-P*syn*-*aaDC*_*Rg*/*Ba*/*Cs*_ were grown in CGXII minimal medium containing 40 g L^−^^1^ glucose and 0.5 mM l-phenylalanine. AROM3 EV and AROM3 *tdc*_*Lb*_ were induced by the addition of 1 mM IPTG at the start of cultivation. For each strain, the mean concentrations of l-tyrosine, tyramine, tryptamine, and 2-phenethylamine (PEA) measured in the culture supernatants are represented by the corresponding area in a pie chart. The total area of each pie correlates with the sum of these measured compounds. Neither l-phenylalanine nor l-tryptophan was detected in any of the culture supernatants (detection limit: 0.1 mM)

Tyramine production was observed for all strains expressing the different decarboxylase genes (Fig. 4), while the empty vector carrying strain produced l-tyrosine (1.7 g L^−1^), but no tyramine. By contrast, the strains AROM3 *aaDC*_*Ba*_, AROM3 *aaDC*_*Cs*_, and AROM3 *aaDC*_*Rg*_, produced tryptamine and 2-phenethylamine (2-PEA) in concentrations up to 0.1 g L^−1^. Notably, neither of these two amines was detected in the AROM3 *tdc*_*Lb*_ culture supernatants.

The highest tyramine titer of 1.9 ± 0.1 g L^−1^ was achieved with AROM3 *tdc*_*Lb*_, followed by AROM3 *aaDC*_*Rg*_ (1.3 ± 0.1 g L^−1^), AROM3 *aaDC*_*Ba*_ (0.8 ± 0.1 g L^−1^), and AROM3 *aaDC*_*Cs*_ (0.2 ± 0.1 g L^−1^). Notably, AROM3 *tdc*_*Lb*_ accumulated 1.9 ± 0.1 g L^−1^ tyramine (and 0.1 ± 0.1 g L^−1^ l-tyrosine), which compared to the l-tyrosine production of AROM3 EV (1.7 ± 0.1 g L^−1^) was about 12% higher by weight, which is equivalent to an increase by approximately 43 mol-%. Thus, tyramine production by AROM3 *tdc*_*Lb*_ benefitted from a metabolic pull caused by the efficient decarboxylation of l-tyrosine to tyramine catalyzed by Tdc_*Lb*_. To the best of our knowledge, this is the first demonstration of fermentative de novo production of tyramine by *C. glutamicum*.

### Tyramine production from alternative carbon sources

The increasing demand for the use of alternative sustainable feedstocks in biotechnological processes prompted us to test if the successful glucose-based de novo production of tyramine by AROM3 *tdc*_*Lb*_ could be transferred to tyramine production based on the pentose sugars ribose or xylose. While *C. glutamicum* is naturally able to grow with ribose, it cannot utilize xylose as a carbon source. Previously, the combined overexpression of the xylose isomerase gene from *Xanthomonas campestris* (*xylA*_*Xc*_) and the endogenous xylulose kinase gene *xylB*_*Cg*_ enabled efficient growth and amino acid production by *C. glutamicum* from xylose (Meiswinkel et al. 2013). Thus, AROM3 *tdc*_*Lb*_ was transformed with pVWEx1-*xylA*_*Xc*_-*xylB*_*Cg*_, resulting in the strain AROM3 *tdc*_*Lb*_* xylA*_*Xc*_*B*_*Cg*_. The parental strain AROM3 *tdc*_*Lb*_ and the xylose utilizing strain AROM3 *tdc*_*Lb*_* xylA*_*Xc*_*B*_*Cg*_ were cultivated in CGXII minimal medium containing 40 g L^−1^ ribose or xylose, respectively, for 72 h. The tyramine production on these carbon sources was compared with a control grown on glucose. AROM3 *tdc*_*Lb*_ showed comparable growth on glucose and ribose (Supplementary Fig. S2). After 72 h of cultivation, only minor amounts of ribose remained (0.2 ± 0.1 g L^−1^), and the tyramine production on ribose did not differ significantly from that on glucose. Both conditions resulted in similar titers, volumetric productivities, and product yield coefficients (Table 2).

**Table 2:** Tyramine titers, volumetric productivities, and product per substrate yield coefficients (Y_PS_) of AROM3 *tdc*_*Lb*_ and AROM3 *tdc*_*Lb*_ *xylA*_*Xc*_*B*_*Cg*_ on different carbon sources. The strains were cultivated in CGXII minimal medium containing 40 g L^−1^ glucose, ribose, or xylose as a carbon source as well as 0.5 mM l-phenylalanine for 72 h. Values represent means and standard deviations of triplicate cultivations

Strain	Carbon source	Tyramine titer [g L^−1^]	Volumetric productivity [g L^−1^ h^−1^]	Y_PS_ [g g^−1^ carbon source]
AROM3 *tdc*_*Lb*_	Glucose	1.6 ± 0.1	0.022 ± 0.001	0.040 ± 0.003
AROM3 *tdc*_*Lb*_	Ribose	1.4 ± 0.1	0.019 ± 0.001	0.034 ± 0.003
AROM3 *tdc*_*Lb*_* xylA*_*Xc*_*B*_*Cg*_	Xylose	1.2 ± 0.1	0.016 ± 0.001	0.037 ± 0.001

The growth of AROM3 *tdc*_*Lb*_* xylA*_*Xc*_*B*_*Cg*_ with xylose showed a longer lag phase compared to AROM3 *tdc*_*Lb*_ cultivated with glucose (4.3 ± 0.9 h compared to 1.2 ± 0.7 h) and the growth rate was more than halved, from 0.26 ± 0.04 h^−1^ to 0.11 ± 0.01 h^−1^ (Supplementary Fig. S2). Nevertheless, both the tyramine titer and the volumetric productivity after 72 h of cultivation with xylose were comparable to those of AROM3 *tdc*_*Lb*_ grown with ribose (Table 2). At the end of cultivation, approximately 8.3 ± 0.3 g L^−1^ of xylose remained, resulting in a product on substrate yield for the tyramine production on xylose that exceeded the yield on ribose. These results indicated that both ribose and xylose serve as suitable alternative carbon sources to glucose, allowing for a sustainable tyramine production with the engineered *C.* *glutamicum* strains.

### Bioreactor batch-mode fermentation for the production of tyramine

Since the overexpression of *tdc*_*Lb*_ in AROM3 led to successful tyramine production on the carbon sources glucose and xylose with similar substrate yield coefficients (0.040 ± 0.003 g g^−1^ glucose and 0.037 ± 0.001 g g^−1^ xylose), it was evaluated whether the tyramine production can be upscaled to bioreactor cultivation in a stable manner. AROM3 *tdc*_*Lb*_ and AROM3 *tdc*_*Lb*_* xylA*_*Xc*_*B*_*Cg*_ were cultivated in bioreactors with 1.5 L CGXII minimal medium with 40 g L^−1^ glucose or 40 g L^−1^ xylose, respectively. Since higher biomass formation was expected during bioreactor cultivation, 1 mM l-phenylalanine and l-tryptophan, each, was supplemented to ensure that neither of these amino acids would be growth limiting.

**Fig. 5:**
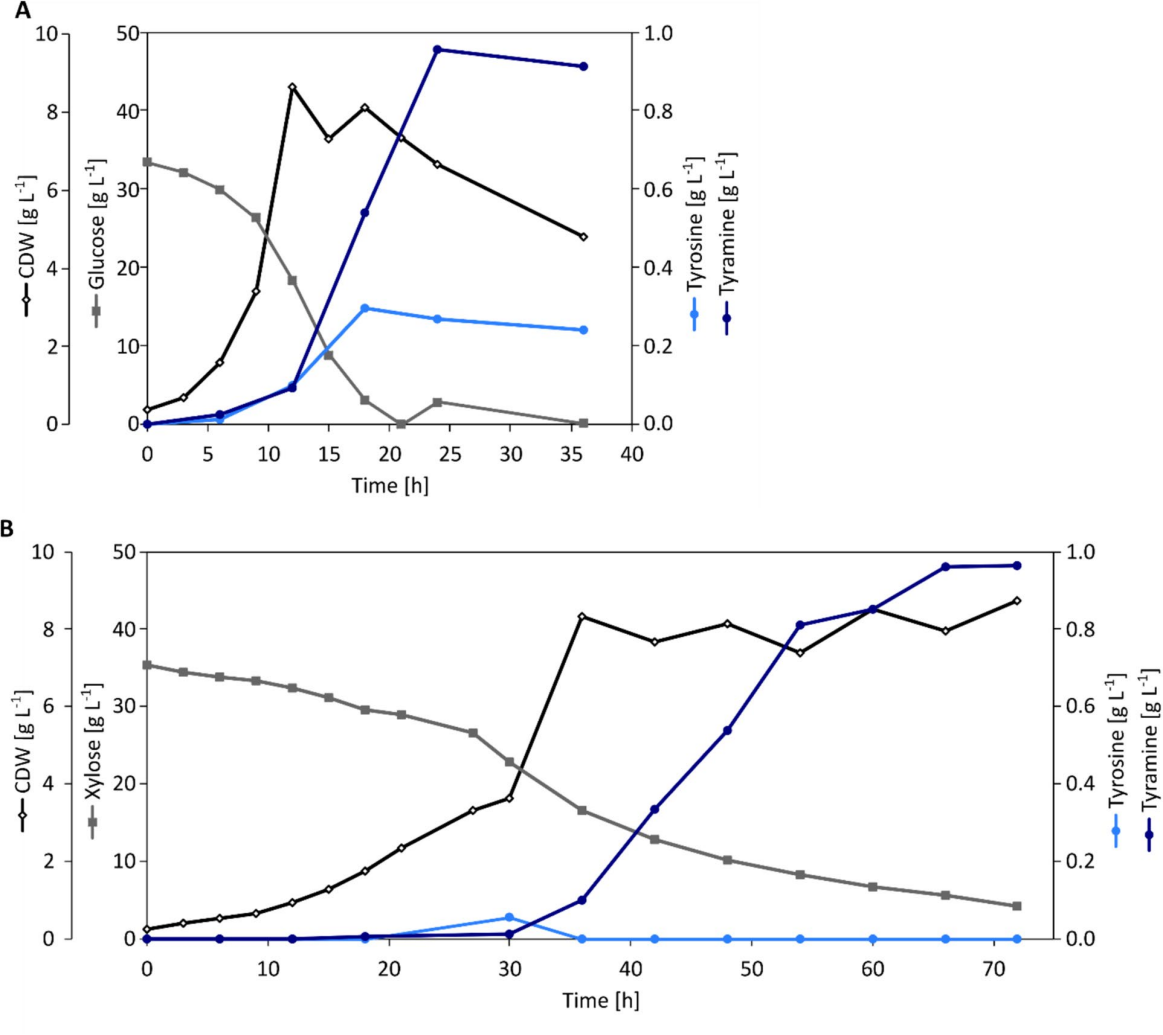
Tyramine production by *C. glutamicum* AROM3 strains expressing *tdcLb* in batch bioreactor cultivation. Strains AROM3 *tdc*_*Lb*_ (A) and AROM3 *tdc*_*Lb*_* xylA*_*Xc*_*B*_*Cg*_ (B) were cultivated in 1.5 L CGXII minimal medium without MOPS containing 40 g L^−1^ of either glucose or xylose as a carbon source, respectively, and supplemented with 1 mM l-phenylalanine, 1 mM l-tryptophan, and 1 mM IPTG. Fermentations were performed with an initial OD_600_ of 1 at 30 °C and pH 7.0 with a constant aeration rate of 0.3 NL min^−^.^1^

In comparison to shake flask cultivation, during which glucose was entirely consumed after 72 h, glucose was consumed after 20 h of fermentation in the bioreactor cultivation. For the strain AROM3 *tdc*_*Lb*_, the maximum CDW of 8.6 g L^−1^was reached after 12 h of fermentation (Fig. 5A), which is approximately 28% higher than that obtained during shake flask cultivation (Supplementary Fig. S2). This higher growth was at the expense of tyramine production, as the final tyramine titer of 1 g L^−1^ corresponded to approximately 63% of the tyramine titers achieved in shake flask cultivation on glucose after 72 h (Table 2). Nevertheless, this tyramine titer was achieved after 24 h of fermentation, resulting in a nearly twofold increase in volumetric productivity of 0.040 g L^−1^ h^−1^ compared to 0.022 g L^−1^ h^−1^ observed for shake flask cultivation. These results indicate that the upscaling to bioreactor cultivation accelerated glucose consumption and tyramine production with AROM3 *tdc*_*Lb*_. However, this was accompanied by the accumulation of 0.27 g L^−1^ l-tyrosine at the end of fermentation, which suggests that the decarboxylation of l-tyrosine by Tdc_*Lb*_ activity became the limiting step during bioreactor cultivation.

Similar to shake flask cultivations, strain AROM3 *tdc*_*Lb*_* xylA*_*Xc*_*B*_*Cg*_ grown in the bioreactor with CGXII minimal medium containing 40 g L^−1^ xylose exhibited slower growth compared to AROM3 *tdc*_*Lb*_ grown on glucose (Fig. 5B), reaching the stationary phase after 36 h. Throughout the fermentation process, l-tyrosine did not accumulate to considerable concentrations, indicating its complete conversion into tyramine. After 72 h of fermentation, tyramine accumulated to a titer of 1 g L^−1^ with a volumetric productivity of 0.013 g L^−1^ h^−1^ and a substrate yield of 0.038 g g^−1^ xylose. These results are comparable to those obtained in shake flask cultivation using xylose as a carbon source (Table 2). Overall, the upscaling of tyramine production on xylose as carbon source to a 1.5 L scale was stable, allowing for a sustainable fermentative tyramine production with AROM3 *tdc*_*Lb*_* xylA*_*Xc*_*B*_*Cg*_.

## Discussion

We have successfully established the de novo production of tyramine in *C. glutamicum* from glucose, ribose, and xylose and demonstrated the transfer to a 1.5 L bioreactor cultivation operated in batch mode*.* Upon overexpression of *tdc* from *L.* *brevis* in the l-tyrosine overproducing strain AROM3, only tyramine was produced, with neither 2-PEA nor tryptamine being detected. In contrast, AROM3 strains overexpressing an AADC gene from either *Bacillus atrophaeus*, *Clostridium sporogenes*, or *Ruminococcus gnavus* produced tryptamine (0.1 g L^−1^) as well as 2-PEA (0.01 g L^−1^) in the case of AROM3 *aaDC*_*Ba,*_ and AROM3 *aaDC*_*Rg*_. Although we cannot exclude differences regarding the expression levels of the AADC genes, our results are consistent with the biochemical characterization of these enzymes as AADC_*Cs*_ and AADC_*Rg*_ have been demonstrated to exhibit up to 1000-fold higher efficiency in decarboxylating l-tryptophan relative to l-tyrosine (Williams et al. 2014). Similarly, AADC_*Ba*_ has been shown to have a 50-fold higher activity for l-tryptophan compared to l-tyrosine (Choi et al. 2021). Conversely, Tdc_*Lb*_ has been characterized to exhibit specificity for l-tyrosine, while accepting neither l-tryptophan nor l-phenylalanine as substrate (Moreno-Arribas and Lonvaud-Funel 2001; Zhang and Ni 2014).

The strain AROM3 *tdc*_*Lb*_, which overexpresses the gene for Tdc from *L.* *brevis*, produced approximately 43 mol-% more tyramine (1.9 ± 0.1 g L^−1^) in shake flasks than the empty vector carrying control strain, AROM3 EV, produced l-tyrosine (1.7 ± 0.1 g L^−1^). This metabolic pull effect could be explained by the decarboxylation of l-tyrosine to tyramine, which reduces the feedback inhibition by this amino acid on its own biosynthesis pathway. This pathway contains three reactions that are known to be inhibited by l-tyrosine. Firstly, the DAHP synthases, which provide DAHP for the shikimate pathway, are feedback-inhibited by all three proteinogenic aromatic amino acids (Hagino and Nakayama 1974a). However, the gene encoding the feedback-inhibition resistant mutant of DAHP synthase from *E. coli* (*aroG*_*Ec*_^fbr^) was integrated into the genome of AROM3. This DAHP synthase mutant was demonstrated to be insensitive to feedback-inhibition by l-phenylalanine concentrations up to 10 mM (Kikuchi et al. 1997). Furthermore, the overexpression of *aroG*_*Ec*_^fbr^ in *E. coli* allowed for a production of approximately 0.2 g L^−1^ l-tyrosine, while no l-tyrosine production was detected for its parental strain (Kim et al. 2018). Therefore, AroG_*Ec*_^fbr^ remains active in AROM3, whereas the native DAHP synthases are inhibited. Furthermore, the activities of the native chorismate mutase (Csm) and the native arogenate dehydrogenase (TyrA) are reduced in vitro to 40% and 50%, respectively, in the presence of 1 mM l-tyrosine (Hagino and Nakayama 1974b, 1975). Because of its decarboxylation, the l-tyrosine concentration in supernatants of the AROM3 *tdc*_*Lb*_ cultures is reduced to concentrations below 0.2 mM after 48 h of shake flask cultivation (Supplementary Fig. S3). It is reasonable to assume that the decarboxylation of l-tyrosine to tyramine results in a similarly low intracellular l-tyrosine concentration. Under these conditions, Csm and TyrA would hardly be subjected to feedback inhibition by l-tyrosine. This would allow for an elevated l-tyrosine biosynthesis, which by subsequent l-tyrosine decarboxylation results in a higher tyramine production.

The de novo tyramine production described here (1.9 ± 0.1 g L^−1^ after 72 h of cultivation) is not yet economically viable since titer, yield and volumetric productivity have to be improved. The addition of yeast extract and tryptone, which generally support better growth and higher production titers in biotechnology, but also significantly increase costs (Sikder et al. 2012), boosted an *E. coli* process (Yang et al. 2022) to a comparable performance (1.965 ± 0.205 g L^−1^ after 72 h of cultivation) as our *C. glutamicum* process. However, as compared to this and other biotechnological approaches, our study stands out, as it does not require yeast extract and tryptone.

Given that l-tyrosine was completely decarboxylated to tyramine by AROM3 *tdc*_*Lb*_ at the end of shake flask cultivation as well as bioreactor cultivation on xylose, it can be concluded that the l-tyrosine synthesis represents the bottleneck under these conditions. Consequently, optimizing the l-tyrosine supply is essential for achieving higher tyramine titers in the future. In *C. glutamicum*, l-tyrosine is synthesized via the l-arogenate pathway, which involves the transamination of prephenate to l-arogenate, followed by its decarboxylation to l-tyrosine (Fazel and Jensen 1979). In several bacteria, including *E. coli*, l-tyrosine is synthesized via the 4-hydroxyphenylpyruvate (4-OHPP) pathway, where the order of decarboxylation and transamination is reversed as compared to the l-arogenate pathway (Koch et al. 1971; Gelfand and Steinberg 1977). Overexpressing the *E. coli* 4-OHPP pathway enzyme genes *tyrA*_*Ec*_ and *tyrB*_*Ec*_, in addition to the native l-tyrosine synthesis pathway in AROM3 *tdc*_*Lb*_, would result in a bifurcated pathway (in parallel via l-arogenate and via 4-OHPP) potentially increasing l-tyrosine formation. A pronounced increase in tyramine concentrations was observed after 12 h and 36 h of bioreactor cultivation on glucose and xylose, respectively. This correlated with the time when the supplemented l-phenylalanine and l-tryptophan were entirely consumed for both bioreactor cultivations. This suggests that, despite the overexpression of the *E. coli aroG*_*Ec*_^fbr^, l-tyrosine production was inhibited by the supplemented amino acids. A potential explanation for this observation is that AroG_*Ec*_^fbr^ is unable to sufficiently compensate for the native DAHP synthases that are inhibited by the supplemented amino acids. The transcription of the DAHP synthase gene *aroF* was demonstrated to be attenuated by *aroR* in the presence of l-phenylalanine (Neshat et al. 2014). Consequently, a potential strategy to mitigate the inhibitory effects on l-tyrosine biosynthesis could involve the mutation of the *aroR* stop codon to prevent reduction in *aroF* expression in the presence of l-phenylalanine and probably also l-tyrosine. To additionally alleviate l-tyrosine biosynthesis from feedback inhibition, endogenous Csm and TyrA mutants resistant to feedback-inhibition might/could be identified in a screening approach similar to the identification of a feedback-inhibition resistant mutant of the chorismate mutase/prephenate dehydrogenase TyrA_*Ec*_^fbr^ from *E. coli* (Lütke-Eversloh and Stephanopoulos 2005).

A further optimized l-tyrosine supply in AROM3 *tdc*_*Lb*_ might result in the decarboxylation step becoming rate limiting for tyramine production. A first indication is the finding that AROM3 *tdc*_*Lb*_ grown with glucose in the bioreactor culture showed a faster glucose consumption and tyramine production, but accumulated l-tyrosine as by-product (Fig. 5). To avoid an incomplete conversion of l-tyrosine upon faster glucose consumption, its decarboxylation should be improved in the future. A number of bacterial decarboxylases assist in maintaining pH homeostasis under acidic stress, since decarboxylation is associated with H^+^-ion consumption and consequently display elevated activity at low pH (Bearson et al. 1997; Viala et al. 2011). For instance, Tdc_*Lb*_ exhibits the highest activity at pH 5 (Zhang and Ni 2014). Accordingly, a two-phase production process, similar to that established for γ-aminobutyric acid (GABA) production with *Bacillus methanolicus* (Irla et al. 2017), comprising an initial phase at pH 7 for cell growth and l-tyrosine accumulation, followed by a second phase at a reduced pH for its decarboxylation into tyramine, may prove advantageous to facilitate tyramine production. Alternatively, bioinformatics and protein engineering could be combined to predict and screen for Tdc_*Lb*_ mutants with enhanced activity at neutral pH conditions (Suplatov et al. 2015).

Another potential avenue for enhancing tyramine production is transport engineering. For example, overexpression of the endogenous aromatic amino acid importer gene *aroP* to increase the reuptake of excreted l-tyrosine (Wehrmann et al. 1995) may increase tyramine production, especially regarding the glucose bioreactor fermentation, during which l-tyrosine accumulated in the culture. Furthermore, the identification and overexpression of the tyramine exporter gene represents another promising strategy for enhancing tyramine production in the future. Currently, a tyramine transporter is unknown, however, it is reasonable to assume that AroP is not responsible, given that the amino acids l-lysine and l-ornithine do have transporters that differ from the transporters of their corresponding decarboxylation products cadaverine and putrescine (Pérez-García and Wendisch 2018).

The division of labor is frequently discussed in the context of the application of engineered microbial consortia for biotechnological production (Sgobba and Wendisch 2020). This is due to the fact that the distribution of different tasks of bioprocesses among different populations reduces the metabolic burden of each individual population. Consequently, the strategic engineering of microbial consortia has the potential to result in the development of more efficient bioprocesses when compared to a single population (Zhou et al. 2015; Tsoi et al. 2018). A consortium of two genetically engineered *E. coli* strains has recently been established for cadaverine production. One strain was genetically modified to overproduce l-lysine, while the other strain decarboxylated l-lysine into cadaverine. This prevented feedback inhibition due to high intracellular cadaverine concentrations in the l-lysine overproducer (Wang et al. 2018). It is conceivable to use consortia for tyramine production, e.g., by co-cultivation of *C. glutamicum* AROM3, which overproduces l-tyrosine, and an *L. brevis* strain that decarboxylates l-tyrosine to tyramine. However, the solubility of l-tyrosine in water is less than 0.5 g L^−1^. Therefore, the l-tyrosine secreted by AROM3 into the medium would rapidly precipitate. After uptake of the dissolved l-tyrosine by *L. brevis* and its decarboxylation into tyramine, l-tyrosine would be dissolved again. However, this process would require time, making it beneficial to combine l-tyrosine production and its decarboxylation in one organism rather than a consortium as done here.

The engineering of AROM3 *tdc*_*Lb*_ for the utilization of ribose and xylose as alternative carbon sources resulted in g tyramine per g substrate yields comparable to those observed for the tyramine production on glucose. Moreover, the upscaling of the xylose-based tyramine production to the 1.5 L scale was demonstrated to be stable, allowing for a sustainable fermentative production of tyramine. As a potential avenue for further optimization, a fed-batch tyramine production process could be established in the future with the aim of achieving higher tyramine titers. Moreover, an evolved *xylA*_*Xc*_*B*_*Cg*_ module with a duplication of the 5′ untranslated region and the beginning of the *xylA*_*Xc*_ gene was demonstrated to improve the growth of *C. glutamicum* on xylose (Werner et al. 2023). The genomic integration of this module into AROM3 *tdc*_*Lb*_ could alleviate the plasmid burden. By extending the substrate spectrum of AROM3 *tdc*_*Lb*_* xylA*_*Xc*_*B*_*Cg*_ to arabinose in addition to xylose, it would be possible to utilize hydrolysates of lignocellulosic agricultural wastes, which contain high amounts of these two pentoses, in more efficient manner (Aristidou and Penttilä 2000). For instance, xylose and arabinose collectively comprise approximately 50% (w/w) of all carbohydrates in rice straw or wheat bran hydrolysates (Gopinath et al. 2011). Another avenue for sustainable tyramine production from waste material is a consortium with a partner that is capable of degrading sustainable feedstocks. Potential partners for this include *Trichoderma reesei* or an α-amylase secreting *E. coli* strain, which allow for the utilization of microcrystalline cellulose and alkaline pretreated corn stover or starch. These have recently been demonstrated to be suitable consortium partners in sustainable biotechnological processes (Scholz et al. 2018; Sgobba et al. 2018).

Biotransformation of l-tyrosine to tyramine is another option for sustainable tyramine production from food wastes with a high protein content (Wohlgemuth 2022). When AROM3 *tdc*_*Lb*_ is cultivated on waste materials with a high l-tyrosine content, the l-tyrosine can be imported into the cell and, in addition to the metabolically produced l-tyrosine, be decarboxylated into tyramine. Potential waste materials for this approach include orange peel, brewing cake, and pumpkin kernel cake, which contain 2 to 14 mg l-tyrosine per g dry matter (Prandi et al. 2019). Notably, an orange peel hydrolysate containing 34 mg L^−1^ l-tyrosine successfully supported growth and l-tyrosine production by *C. glutamicum* AROM3 (Junker et al. 2024). Orange peel and brewing cake accumulate to a considerable extent, amounting up to 20 million tons per year (Thiago et al. 2014; Aboagye et al. 2017). Also, pumpkin kernel cake is abundant with approximately 11,500 tons being produced annually as a by-product of pumpkin seed oil production (Balbino et al. 2019).

Consequently, with the metabolic optimization described above and the utilization of agricultural side streams, the proof-of-concept of sustainable tyramine production by *C. glutamicum* developed in this study might lead to an economically viable and environmentally friendly sustainable tyramine production process in the near future. Moreover, the engineered *C. glutamicum* strain may be used for extension of the tyramine production pathway by acetylation or hydroxylation steps to yield *N-*acetyltyramine or dopamine, respectively (Chenprakhon et al. 2017; Pan et al. 2023), or to obtain its anti-inflammatory derivative tyrosol (Satoh et al. 2012).

## Supplementary Information

Below is the link to the electronic supplementary material.

Supplementary file1 (DOCX 185 KB)

## Data Availability

All data are present in the manuscript and its Supplement.
