# Supplementary material for: Sustainable production of the drug precursor tyramine by engineered Corynebacterium glutamicum
Source: Appl Microbiol Biotechnol. 2024 Oct 30;108:499. doi: 10.1007/s00253-024-13319-8 (PMC11525245; doi:10.1007/s00253-024-13319-8)
Supplement: Supplementary file 1 [file 253_2024_13319_MOESM1_ESM.docx]

Supplementary material to Applied Microbiology and Biotechnology

# Sustainable production of the drug precursor tyramine by engineered *Corynebacterium glutamicum*

Sara-Sophie Poethe^1✝^, Nora Junker^1✝^, Florian Meyer^1^, Volker F. Wendisch^1^*

^1^Genetics of Prokaryotes, Faculty of Biology and CeBiTec, Bielefeld University, Universitätsstr. 25, 33615 Bielefeld, Germany; sara-sophie.poethe@uni-bielefeld.de (S-S.P; ORCID 0000-0001-6697-7971); nora.junker@uni-bielefeld.de (N.J.; ORCID 0009-0004-6850-1167); [florian.meyer@uni-bielefeld.de](mailto:florian.meyer@uni-bielefeld.de) (F.M.; ORCID 0000-0002-8349-1153)

***** Correspondence: volker.wendisch@uni-bielefeld.de (V.F.W.); Tel.: +49-521-106-5611; ORCID 0000-0003-3473-0012

^✝ These authors contributed equally to this work^

**Tab. S1 Oligonucleotides used in this work**

| **Purpose/ Target** | **Primer name** | **Sequence**  **(5’ → 3’)** |
| --- | --- | --- |
| Verification of genes integrated into pECXT-P*syn* | pECXT_Prytt_fw | TCAGTGAGCGAGGAAGC |
|  | pECXT_rv | TACTGCCGCCAGGCAAATTC |
| Gibson assembly cloning of *tdc_Lb_* into pECXT99A | tdc_pECXT99A_fw | CAGGAAACAGACCATGGAGCGTAAGCACCATTTTACATTCAAAAGGGGTTATTTTATGGAAAAATCCAACCG |
|  | tdc_pECXT99A_rv | GAGGATCCCCGGGTACCGAGCTCGAATTTTACACGTTTTCCTTCTGGTTC |
| Verification of genes integrated into pECXT99A | pECXT99A_fw | TTTGCGCCGACATCATAACGGTTCTG |
|  | pECXT99A_rv | CTACGGCGTTTCACTTCTGAGTTCGG |
| Sequencing of *tdc_Lb_* | tdc_Seq1_fw | CGGCCGTTACTGGGGCCATATGAACTC |
|  | tdc_Seq2_rv | GCTTTGTACGCCGCGTAAACTTC |
|  | tdc_Seq3_fw | CAGAAGTTTACGCGGCGTACAAAG |
|  | tdc_RBS_Seq_rv | CAGAACCGTCTTCATGTGTCCC |
| Verification of genes integrated into pVWEx4 | Ptac_fw | CGCTTCCACTTTTTCCCGCGT |
|  | term_rv | GCATTTATCAGGGTTATTGTC |

**Tab. S2 Sequence of codon-harmonized *tdc_Lb_*, its optimized RBS (underlined), and translational start and stop codons (bold)**

| **Gene name** | **Sequence (5’ → 3’)** |
| --- | --- |
| *tdc_Lb_* | GCGTAAGCCCATTCATAAGGGGTTATTTT**ATG**GAAAAATCCAACCGTTCTCTAAAAGATCTGGATCTGAACGCGCTGTTCATCGGAGATAAGGCAGAAAACGGACAACTGTACAAAGATCTTCTAAACAAACTGGTCGATGAACACCTGGGATGGCGTAAGAATTATATACCCTCTGATCCAAACATGATCGGCCCAGAAGATCAGAATTCACCAGCGTTCAAAAAGACTGTGGGACACATGAAGACGGTTCTGGATCAGCTGTCTGAACGCATCCGTACGGAGTCAGTGCCATGGCACTCGGCCGGCCGTTACTGGGGCCATATGAACTCTGAGACTCTAATGCCCGCCCTACTGGCGTACAATTACGCCATGCTGTGGAACGGCAACAATGTCGCCTACGAATCATCTCCAGCGACCTCGCAGATGGAAGAAGAAGTGGGTCAGGAATTCGCACGTCTGATGGGCTACGACTACGGTTGGGGTCATATCGTTGCGGATGGCTCGCTGGCCAACCTAGAGGGACTATGGTACGCGCGTAACATCAAATCTCTACCTTTCGCAATGAAAGAAGTGAACCCAGAACTGGTGGCAGGCAAGTCGGATTGGGAGCTACTAAACATGCCTACTAAAGAAATCATGGATCTACTGGAGAACGCGGGCTCACAAATTGATGAAGTTAAGAAGCGTTCTGCCCGTTCCGGCAAGAACCTACAGCGTCTAGGAAAATGGCTAGTACCACAGACCAAGCACTACTCATGGATGAAGGCAGCCGATATTATCGGCATCGGCCTGGATCAGGTGGTGCCCGTGCCAATCGATTCCAACTACCGCATGGATATCCAGGCACTGGAATCCATCATCCGTAAATACGCGGCCGAAAAGACGCCAATACTAGGTGTCGTGGGCGTCGCAGGATCTACTGAAGAAGGCGCAGTGGATGGTATCGATAAGATCGTTGCCCTGCGTCAGAAGCTTCAGAAGGAAGGAATCTATTTTTACCTACATGTAGATGCCGCGTACGGCGGATACGCCCGCGCGCTGTTTCTGGACGAGGACGATCAATTCATCCCATATAAAAACCTGCAGAAAGTACACGCGGAAAACCACGTTTTTACCGAAGATAAAGAATATATTAAACCAGAAGTTTACGCGGCGTACAAAGCCTTTGATCAGGCGGAGTCGATCACGATCGATCCCCACAAGATGGGATACGTACCATATTCGGCCGGAGGTATCGTTATCCAGGATATCCGCATGCGTGACACCATCTCGTACTTCGCGACGTACGTATTCGAGAAGGGCGCAGATATCCCTGCGCTGCTAGGCGCCTACATCCTTGAGGGTTCGAAAGCGGGCGCAACTGCCGCGTCAGTGTGGGCGGCGCATCATACGCTGCCACTGAATGTCACGGGATACGGAAAGCTGGAAGGCGCATCTATCGAAGGAGCCCATCGTTATTACGATTTTCTGAAGAACCTGAAGTTCGAAGTCGCCGGCAAACGCATCTCTGTGCACCCTCTGATTTCTCCCGACTTTAACATGGTGGACTACGTGCTGAAAGAAGATGGTAACGATGACCTGATCGAAATGAACCGTCTGAACCACGCATTTTACGAACAGGCGTCATACGTGAAAGGATCGCTGTACGGCAAAGAATACATTGTATCTCACACCGACTTCGCCATTCCAGATTACGGCGATTCCCCACTGGCGTTCGTGGAATCCCTAGGTTTCAGCGAAGTGGAATGGCGTCACGCAGGAAAGGTGACGATTATCCGTGCCTCGGTGATGACCCCTTACATGAACCAGCGTGAAAATTTCGACTATTTCGCGCCACGTATTAAAAAAGCGATCCAGGCGGACCTAGAAAAAGTTTACGCCTCGGTGAACCAGAAGGAAAACGTG**TAA** |

**Tab. S3 HPLC solvent gradient and flow rate protocol used for the analysis of l-tyrosine, l-tryptophan, l-phenylalanine, and their corresponding amines**

| **Time**  **[min]** | **Ratio solvent B**  **[%]** | **Flow**  **[mL min^-1^]** |
| --- | --- | --- |
| 0.0 | 20 | 0.7 |
| 1.0 | 38 | 0.7 |
| 3.0 | 46 | 0.7 |
| 5.0 | 50 | 0.5 |
| 5.5 | 51 | 0.5 |
| 8.3 | 53 | 0.5 |
| 9.0 | 54 | 0.5 |
| 12.0 | 58 | 0.8 |
| 16.0 | 65 | 1.0 |
| 19.0 | 80 | 1.2 |
| 19.5 | 90 | 1.2 |
| 20.5 | 20 | 1.2 |
| 22.5 | 20 | 1.2 |

**Fig. S1 Degradation test of tyramine by *C. glutamicum* AROM3.** Tyramine concentrations determined via HPLC measurement for the AROM3 cultures at the beginning and end of cultivation in 10 mL CGXII minimal medium supplemented with 40 g L^-1^ glucose, 0.5 mM l-phenylalanine, and 0-50 mM tyramine for 48 h. Bars and error bars represent means and standard deviations of triplicate cultivations. Grey circles represent the values measured for the triplicates. Significance was calculated with a two-sided Student’s t-test with n.s.: not significant

**Tab. S4 List of labels for enzymes depicted in Fig. 2 with the corresponding Swiss-Prot identifiers, annotations, organisms, and amino acid sequence identities to Tdc*_Lb_***

| **Label** | **Identifier** | **Annotation** | **Organism** | **Identity [%]** |
| --- | --- | --- | --- | --- |
| TYRDC_LEVBR | SP:J7GQ11 | l-tyrosine decarboxylase | *Levilactobacillus brevis* | 100 |
| TYRDC_ENTFA | SP:Q838D6 | l-tyrosine decarboxylase | *Enterococcus faecalis* (strain ATCC 700802 / V583) | 74.6 |
| TYRDC_ENTF3 | SP:P0DTQ4 | l-tyrosine decarboxylase | *Enterococcus faecalis* (strain EnGen0310 / MMH594) | 74.6 |
| TYRDC_ENTFC | SP:A0A481NV25 | l-tyrosine decarboxylase | *Enterococcus faecium* | 60.2 |
| PANP_ALIF1 | SP:Q5E6F9 | Aspartate 1-decarboxylase | *Aliivibrio fischeri* (strain ATCC 700601 / ES114) | 27 |
| MFNA_METM7 | SP:A6VIC0 | Probable l-tyrosine/l-aspartate decarboxylase | *Methanococcus maripaludis* (strain C7 / ATCC BAA-1331) | 28.3 |
| MFNA_METM5 | SP:A4G060 | Probable l-tyrosine/l-aspartate decarboxylase | *Methanococcus maripaludis* (strain C5 / ATCC BAA-1333) | 29.1 |
| MFNA_METTH | SP:O27188 | Probable l-tyrosine/l-aspartate decarboxylase | *Methanothermobacter thermautotrophicus* (strain ATCC 29096 / DSM 1053 / JCM 10044 / NBRC 100330 / Delta H) | 27.4 |
| MFNA_METKA | SP:Q8TV92 | Probable l-tyrosine/l-aspartate decarboxylase | *Methanopyrus kandleri* (strain AV19 / DSM 6324 / JCM 9639 / NBRC 100938) | 30.7 |
| MFNA_METVS | SP:A6URB4 | Probable l-tyrosine/l-aspartate decarboxylase | *Methanococcus vannielii* (strain ATCC 35089 / DSM 1224 / JCM 13029 / OCM 148 / SB) | 29 |
| MFNA_METMP | SP:Q6M0Y7 | Probable l-tyrosine/l-aspartate decarboxylase | *Methanococcus maripaludis* (strain S2 / LL) | 28 |
| MFNA_METS3 | SP:A5ULW4 | Probable l-tyrosine/l-aspartate decarboxylase | *Methanobrevibacter smithii* (strain ATCC 35061 / DSM 861 / OCM 144 / PS) | 26.8 |
| MFNA_THEKO | SP:Q5JJ82 | l-aspartate decarboxylase | *Thermococcus kodakarensis* (strain ATCC BAA-918 / JCM 12380 / KOD1) | 24.8 |
| MFNA_PYRFU | SP:Q8U1P6 | Probable l-aspartate decarboxylase | *Pyrococcus furiosus* (strain ATCC 43587 / DSM 3638 / JCM 8422 / Vc1) | 26.5 |
| MFNA_PYRAB | SP:Q9UZD5 | Probable l-aspartate decarboxylase | *Pyrococcus abyssi* (strain GE5 / Orsay) | 25.7 |
| MFNA_META3 | SP:A6UVR4 | Probable l-tyrosine/l-aspartate decarboxylase | *Methanococcus aeolicus* (strain ATCC BAA-1280 / DSM 17508 / OCM 812 / Nankai-3) | 25.4 |
| MFNA_METAC | SP:Q8TUQ9 | Probable l-tyrosine/l-aspartate decarboxylase | *Methanosarcina acetivorans* (strain ATCC 35395 / DSM 2834 / JCM 12185 / C2A) | 26.6 |
| MFNA_METJA | SP:Q60358 | l-tyrosine/l-aspartate decarboxylase | *Methanocaldococcus jannaschii* (strain ATCC 43067 / DSM 2661 / JAL-1 / JCM 10045 / NBRC 100440) | 27.3 |
| MFNA_METMA | SP:Q8PXA5 | Probable l-tyrosine/l-aspartate decarboxylase | *Methanosarcina mazei* (strain ATCC BAA-159 / DSM 3647 / Goe1 / Go1 / JCM 11833 / OCM 88) | 27.1 |
| MFNA_PYRHO | SP:O58679 | l-aspartate/l-glutamate decarboxylase | *Pyrococcus horikoshii* (strain ATCC 700860 / DSM 12428 / JCM 9974 / NBRC 100139 / OT-3) | 25.9 |
| MFNA_THEGJ | SP:C5A2X8 | Probable l-aspartate decarboxylase | *Thermococcus gammatolerans* (strain DSM 15229 / JCM 11827 / EJ3) | 25.2 |
| MFNA_METTP | SP:A0B9M9 | Probable l-tyrosine/l-aspartate decarboxylase | *Methanothrix thermoacetophila* (strain DSM 6194 / JCM 14653 / NBRC 101360 / PT) | 27.7 |
| MFNA_METBF | SP:Q46DU3 | Probable l-tyrosine/l-aspartate decarboxylase | *Methanosarcina barkeri* (strain Fusaro / DSM 804) | 27.5 |
| MFNA_METAR | SP:Q0W498 | Probable l-tyrosine/l-aspartate decarboxylase | *Methanocella arvoryzae* (strain DSM 22066 / NBRC 105507 / MRE50) | 26 |
| MFNA_METPE | SP:B8GDM7 | Probable l-tyrosine/l-aspartate decarboxylase | *Methanosphaerula palustris* (strain ATCC BAA-1556 / DSM 19958 / E1-9c) | 27.5 |
| DDC_HAEIN | SP:P71362 | l-2,4-diaminobutyrate decarboxylase | *Haemophilus influenzae* (strain ATCC 51907 / DSM 11121 / KW20 / Rd) | 24.5 |
| MFNA_ARCFU | SP:O28275 | Probable l-aspartate decarboxylase | *Archaeoglobus fulgidus* (strain ATCC 49558 / DSM 4304 / JCM 9628 / NBRC 100126 / VC-16) | 25.3 |
| MFNA_METST | SP:Q2NHY7 | Probable l-tyrosine/l-aspartate decarboxylase | *Methanosphaera stadtmanae* (strain ATCC 43021 / DSM 3091 / JCM 11832 / MCB-3) | 24.3 |
| MFNA_METHJ | SP:Q2FSD2 | Probable l-tyrosine/l-aspartate decarboxylase | *Methanospirillum hungatei* JF-1 (strain ATCC 27890 / DSM 864 / NBRC 100397 / JF-1) | 27.7 |
| MFNA_HALSA | SP:Q9HSA3 | Probable l-aspartate decarboxylase | *Halobacterium salinarum* (strain ATCC 700922 / JCM 11081 / NRC-1) | 24.6 |
| MFNA_HALS3 | SP:B0R349 | Probable l-aspartate decarboxylase | *Halobacterium salinarum* (strain ATCC 29341 / DSM 671 / R1) | 24.6 |
| DDC_ACIBA | SP:Q43908 | l-2,4-diaminobutyrate decarboxylase | *Acinetobacter baumannii* | 22.9 |
| MFNA_HALMA | SP:Q5V1B4 | Probable l-aspartate decarboxylase | *Haloarcula marismortui* (strain ATCC 43049 / DSM 3752 / JCM 8966 / VKM B-1809) | 28.4 |
| MFNA_METMJ | SP:A3CWM4 | Probable l-tyrosine/l-aspartate decarboxylase | *Methanoculleus marisnigri* (strain ATCC 35101 / DSM 1498 / JR1) | 25 |
| MFNA_METBU | SP:Q12VA2 | Probable l-tyrosine/l-aspartate decarboxylase | *Methanococcoides burtonii* (strain DSM 6242 / NBRC 107633 / OCM 468 / ACE-M) | 23.2 |
| RHBB_RHIME | SP:Q9Z3R1 | l-2,4-diaminobutyrate decarboxylase | *Rhizobium meliloti* (strain 1021) | 26.7 |
| BUT83_GIBZE | SP:I1RV23 | Glutamate decarboxylase-like protein FG08083 | *Gibberella zeae* (strain ATCC MYA-4620 / CBS 123657 / FGSC 9075 / NRRL 31084 / PH-1) | 22.4 |
| MFNA_NATPD | SP:Q3IT46 | Probable l-aspartate decarboxylase | *Natronomonas pharaonis* (strain ATCC 35678 / DSM 2160 / CIP 103997 / JCM 8858 / NBRC 14720 / NCIMB 2260 / Gabara) | 27.9 |
| MFNA_METB6 | SP:A7IAB9 | Probable l-tyrosine/l-aspartate decarboxylase | *Methanoregula boonei* (strain DSM 21154 / JCM 14090 / 6A8) | 24.3 |
| MFNA_METLZ | SP:A2STQ3 | Probable l-tyrosine/l-aspartate decarboxylase | *Methanocorpusculum labreanum* (strain ATCC 43576 / DSM 4855 / Z) | 25.3 |
| TRPDC_CLOS1 | SP:J7SZ64 | Tryptophan decarboxylase | *Clostridium sporogenes* (strain ATCC 15579) | 25.5 |
| CSAD_MOUSE | SP:Q9DBE0 | Cysteine sulfinic acid decarboxylase | *Mus musculus* | 27.3 |
| DTXS4_METRA | SP:E9FCP7 | l-aspartate decarboxylase dtxS4 | *Metarhizium robertsii* (strain ARSEF 23 / ATCC MYA-3075) | 29.1 |
| CSAD_RAT | SP:Q64611 | Cysteine sulfinic acid decarboxylase | *Rattus norvegicus* | 26.9 |
| AADC_BACAT | SP:I0DFJ0 | Aromatic-l-amino-acid decarboxylase | *Bacillus atrophaeus* | 24.5 |
| GADL1_XENTR | SP:Q28D99 | Acidic amino acid decarboxylase GADL1 (Fragment) | *Xenopus tropicalis* | 21.1 |
| SGPL1_PONAB | SP:Q5R4G0 | Sphingosine-1-phosphate lyase 1 | *Pongo abelii* | 23.4 |
| TRPDC_RUMGV | SP:A7B1V0 | Tryptophan decarboxylase | *Ruminococcus gnavus* (strain ATCC 29149 / VPI C7-9) | 24.4 |
| SGPL1_MOUSE | SP:Q8R0X7 | Sphingosine-1-phosphate lyase 1 | *Mus musculus* | 24.5 |
| CSAD_HUMAN | SP:Q9Y600 | Cysteine sulfinic acid decarboxylase | *Homo sapiens* | 26.4 |

**Fig. S2 Growth of *C. glutamicum* AROM3 *tdc_Lb_* and AROM3 *tdc_Lb_ xylA_Xc_B_Cg_* on glucose, ribose, and xylose as carbon sources.** Growth curves (A) of AROM3 *tdc_Lb_* cultivated in CGXII minimal medium containing 40 g L^-1^ of either glucose (blue circles with dashed line) or ribose (green squares with straight line) as well as of AROM3 *tdc_Lb_ xylA_Xc_B_Cg_* cultivated on CGXII minimal medium containing containing 40 g L^‑1^ xylose (pink rectangles with dashed line). Growth rate (grey) and lag phase (red) are depicted in B. Values represent means and standard deviations of triplicate cultivations. Significance was calculated with a two-sided Student’s t-test with n.s.: p > 0.05, *: p < 0.05, **: p < 0.01

**Fig. S3 Time course of the production of tyramine and tyrosine by *C. glutamicum* AROM3 *tdc_Lb_*.** AROM3 *tdc_Lb_* was grown in CGXII minimal medium containing 40 g L^‑1^ glucose, 0.5 mM l‑phenylalanine, and 1 mM IPTG. Values represent means and standard deviations of tyramine (filled dark blue circles with straight line) and tyrosine (light blue squares with dashed line) concentrations measured via HPLC for triplicate cultivations.
